# Validation of Reference Genes for Gene Expression Studies by RT-qPCR in HepaRG Cells during Toxicity Testing and Disease Modelling

**DOI:** 10.3390/cells9030770

**Published:** 2020-03-21

**Authors:** Joanna Brzeszczyńska, Filip Brzeszczyński, Kay Samuel, Katie Morgan, Steven D. Morley, John N. Plevris, Peter C. Hayes

**Affiliations:** 1 University of Edinburgh Hepatology Laboratory, Chancellor’s Building, Edinburgh BioQuarter, 49 Little France Crescent, Edinburgh EH16 4SB, UK; Katie.Morgan@ed.ac.uk (K.M.); Steve.Morley@ed.ac.uk (S.D.M.); J.Plevris@ed.ac.uk (J.N.P.); P.Hayes@ed.ac.uk (P.C.H.); 2 Department of Molecular Biophysics, University of Lodz, 90-236 Lodz, Poland; 3 School of Medicine, Dentistry and Biomedical Sciences, Queen’s University Belfast, 97 Lisburn Rd, Belfast BT9 7BL, UK; ff.brzeszczynski@gmail.com; 4 Scottish National Blood Transfusion Service, Jack Copland Centre, 52 Research Avenue North, Edinburgh EH14 4BE, UK; K.Samuel@ed.ac.uk

**Keywords:** Reference Genes (RG), HepaRG cells, APAP (Acetaminophen), CPZ (Chlorpromazine)

## Abstract

Gene expression analysis by quantitative real-time polymerase chain reaction (RT-qPCR) is routinely used in biomedical studies. The reproducibility and reliability of the data fundamentally depends on experimental design and data interpretation. Despite the wide application of this assay, there is significant variation in the validation process of gene expression data from research laboratories. Since the validity of results depends on appropriate normalisation, it is crucial to select appropriate reference gene(s), where transcription of the selected gene is unaffected by experimental setting. In this study we have applied geNorm technology to investigate the transcription of 12 ‘housekeeping’ genes for use in the normalisation of RT-qPCR data acquired using a widely accepted HepaRG hepatic cell line in studies examining models of pre-clinical drug testing. geNorm data identified a number of genes unaffected by specific drug treatments and showed that different genes remained invariant in response to different drug treatments, whereas the transcription of ‘classical’ reference genes such as GAPDH (glyceralde- hyde-3-phosphate dehydrogenase) was altered by drug treatment. Comparing data normalised using the reference genes identified by geNorm with normalisation using classical housekeeping genes demonstrated substantial differences in the final results. In light of cell therapy application, RT-qPCR analyses has to be carefully evaluated to accurately interpret data obtained from dynamic cellular models undergoing sequential stages of phenotypic change.

## 1. Introduction

The quantitative real-time polymerase chain reaction (RT-qPCR) analysis of gene expression is an important measure in biomedical research, offering accuracy, rapid analysis, real-time detection, and sensitivity for mRNA transcription profiling. Undoubtedly, its advantage is the detection of a small number of mRNA copies difficult to detect using other techniques. Moreover, because the mRNA molecule links DNA and protein expression, the quantification and analysis of changes in mRNA transcription levels are informative in the study of developmental processes, therapeutic drug testing and treatment, tumorigenesis, and for disease diagnosis. However, there are multiple pitfalls and problems associated with RT-qPCR methodology and analysis that have to be addressed to produce accurate data. These include the standardization of sample preparation/storage, reference gene and control sample selection, primer design, and statistical analysis. There are a few published papers that emphasize that many RT-qPCR experiments suffer from a lack of critical evaluation or are incorrectly designed and difficult to repeat due to poor data quality [1]. One of the major issues recognised in gene transcription analysis is in the selection of reference genes (RG) for the normalisation of data that can result in inaccurate interpretation of results.

Housekeeping gene expression should remain stable across changing conditions within an experiment regardless of tissue type, disease state, and disease progression and/or treatment [2,3]. To date, several publications agree with the finding that the expression of GAPDH, 18S, or β-actin, commonly used for the normalisation of data, vary considerably under different experimental conditions and are consequently unsuitable as reference genes for RNA transcription analysis [4,5,6]. Indeed, almost all genes are modified under some conditions [7] and therefore there is no single gene where the stability of expression can be guaranteed in all studies. The selection of appropriate reference genes for RT-qPCR data normalisation is essential for the reliability and reproducibility of RT-qPCR results and to accurately compensate for variations in experimental conditions, tissue composition or cellularity. To encourage better experimental RT-qPCR practices, it has been recommended that a minimum of three genes with the highest expression stability throughout experimentation should be used as reference genes for data normalization [5].

Human HepaRG cells are a highly differentiated hepatocyte:cholangiocyte, co-culture model, which maintains a stable phenotype for several weeks in culture, including liver-specific functions, intact drug/lipid metabolism, bile-canalicular polarity, and liver-specific cytochrome P450 (CYP) enzyme expression at levels comparable with primary human hepatocytes (PHHs) [8,9,10,11]. Although HepaRG cells are derived from a hepatocellular carcinoma, their karyotyping reveals a stable and unique phenotype [10]. For these reasons, it is expected that the use of HepaRG cells will gain global application as a human-relevant cell model. Ceelen et al. has presented optimal reference genes for gene expression analyses in HepaRG progenitor cells undergoing differentiation with 2% dimethylsulfoxide (DMSO), where significant phenotypic and molecular changes occurred with time [12]. However, similar studies in fully differentiated HepaRG cells maintained in culture have not yet been published.

It has been demonstrated that under different experimental conditions and during differentiation, the analysis of HepaRG RT-qPCR data showed a lack of reproducibility and validity as a properly established normalization method had not been applied [13,14]. This was also seen when iPS cells were differentiated to hepatocyte-like cells [15]. Due to the heterogeneity of cell types in differentiating cultures, reference genes had to be optimised for each sequential stage of differentiation [15]. These observations are in agreement with other surveys of RT-qPCR-based publications [16]. Considering the vast financial and development efforts put into research by pharmaceutical and/or biotechnology companies, and the increasing need to use in vitro HepaRG models as an alternative for primary human hepatocytes in pre-clinical drug testing, there is a necessity to investigate and report normalization methods for RT-qPCR data analyses in the area of drug safety evaluation.

In this study, we have investigated the suitability of 12 commonly used housekeeping genes for use as RT-qPCR normalisers in the fully differentiated HepaRG cell line. The cells were subjected to two different liver hepatotoxins: acetyl-para-aminophenol (APAP), known to affect intercellular tight junctions [17] and chlorpromazine (CPZ), which causes damage resulting in cholestasis [18]. The aim of this study was first, to establish whether expression of ‘classical’ reference genes in the HepaRG cell line were differentially affected by exposure over the same time period to different hepatotoxins. Secondly, to identify stably expressed genes for the normalisation of data from different treatments. Finally, we explored whether there were substantial differences in the results using ‘classical’ reference genes compared to stably expressed genes identified using geNorm for the normalisation of data. Here, we highlight the importance of identifying stable reference genes (RG) in relatively simple and classical experimental conditions when the same cell type is exposed to different molecules.

## 2. Materials and Methods

### 2.1. Cell Culture

The cell culture was carried out according to previously published protocol [9,11,17,19]. HepaRG fully differentiated cells (HPR116-TA08; Cryopreserved HepaRG™ cells; Biopredic Int., Rennes, France) were thawed from liquid nitrogen and plated in Biopredic’s proprietary General Purpose Medium (GPS) without dimethyl sulfoxide (DMSO) for up to 2 days. Since traditional HepaRG cells are terminally differentiated, it was necessary to provide a medium containing DMSO earlier for this culture than the HepaRG progenitor cell line. For this reason, on day 3–4 of culture, the medium was changed to Biopredic’s proprietary Metabolism Maintenance Medium (MMM) containing DMSO. Here, DMSO continues to support the fully differentiated state of the HepaRG cell throughout the life of culture.

HepaRG cells were cultured using specialized media following the supplier’s protocols. Each medium was made up in William’s E Medium with GlutaMAX™ (Sigma), supplemented with appropriate additives (ADD). Cells were seeded at 2.4 × 105/cm^2^ on TCP (tissue culture plate). On day 3, the medium was changed to HepaRG™ Maintenance and Metabolism Medium (MMM; ADD620, containing 2% DMSO), and HepaRGs was cultured to confluence. MMM medium was renewed every 2–3 days. On day 8, HepaRG cells were challenged with either APAP (0 mM, 2.5 mM, 5 mM, 10 mM, or 20 mM A7085, Sigma-Aldrich) or CPZ (0 µM, 25 µM, 50 µM, or 100 µM C8138-5G Sigma-Aldrich) in MMM for 24h, using DMSO-vehicles as controls. Experiments using APAP- and CPZ-treated cells were repeated independently 3 times using three technical replicates in every experiment.

### 2.2. Viability Assays

Throughout the experimental process, differentiated HepaRG cells were regularly assessed for viability according to previously published procedure [17,19]. The cells were seeded on a 96 well standard tissue culture plate and grown to confluency (day 8). After a 24-h treatment with CPZ or APAP, the supernatant was removed and PrestoBlue^®^ (A-13262; Life Technologies, Paisley, UK) was added to cell culture and incubated for 30 min at room temperature. Fluorescence was measured using a GloMax+ Microplate Multimode Reader (Promega, Southampton, UK) at 520 nm per the vendor’s instructions. After treatment with PrestoBlue assay, the cells were washed and lysed using Promega CellTiter-Glo^®^ Luminescent Cell Viability Assay (G7570; Promega, Southampton, UK). After 30 min of incubation at room temperature, the detection agent was added to this luciferase-based assay and Bioluminescent signals were measured with the above GloMax plate reader.

### 2.3. Total RNA Isolation

The cells were harvested for total RNA extraction from untreated HepaRG cells and 24 h after treatment with the different concentrations of drugs. Total RNA was extracted using Aqueous kit columns (Life Technologies, Paisley, UK), as directed by the manufacturer with an on-column DNAse digestion step. RNA was quantified using the Nanodrop instrument (Thermo Fisher). The quality and purity of RNA was examined using 260/280 and 260/230 ratios [20]. The Agilent bioanalyzer (Agilent, UK) was used to assess RNA integrity using previously published protocols [20]. All samples had 260/280 ratios above 1.8 and RIN scores above 7.5.

### 2.4. Real-Time Polymerase Chain Reaction (RT-qPCR)

The nanoScript to RT kit (PrimerDesign, Southampton, UK) was used to convert 1 μg of RNA to cDNA following the manufacturer’s directions. The quantitative validation of the expression of selected genes was performed by RT-qPCR (Roche LightCycler^®^ 96 System) using custom PrimerDesign primers (PrimerDesign geNormTM kit) and applying the PrecisionPLUS RT-qPCRMaster Mix (PrimerDesign, Southampton, UK), following the manufacturer’s protocol. Reactions were run in triplicate on a LightCycler^®^ 96 Instrument. The running conditions were 95 °C for 2 min followed by 40 cycles of 95 °C for 15 s and 60 °C for 60 s. Amplification was performed for each cDNA (25 ng) sample in triplicate. The fold change in expression of the target gene relative to the internal control gene was assessed. The RT-qPCR data were presented as the fold-change in gene expression normalized to the selected common endogenous reference genes and relative to the control. See Table 1 for RT-qPCR primers.

### 2.5. Identification of Suitable Reference Genes

The geNorm RG Selection Kit (PrimerDesign) was used to evaluate expression stability of 12 commonly used RGs in HepaRG control and hepatotoxin treated cells to identify a set of stably expressed RGs to be used in the normalisation of data. The tested RGs are detailed in Table 2. The geNorm output ranked the candidate reference genes according to their expression stability (M). Using this approach, we identified a minimum set of the most stably expressed genes in various experimental settings as RG candidates. These were used for the analysis of the changes in the expression of the genes of interest.

### 2.6. Quality Control on Post-RT-qPCR Data

The quality of RT-qPCR data was carefully investigated during multiple processing steps of RT-qPCR. The samples were treated with DNAase before cDNA synthesis. The assessment of the shape of sigmoidal amplification curves showed reactions of a high quality and an absence of inhibitors. Where the melting curve showed non-specific amplification products, the results were excluded from analysis. The melting curve analysis showed that all primer pairs led to the amplification of the desired product in the different samples.

### 2.7. RT-qPCR Efficiency Evaluation

#### 2.7.1. Determination of Reference Gene Expression Stability

To determine the most suitable RGs to use for the normalisation of the expression of the genes of interest in different drug-treated groups, the geNorm M value (M) and coefficient of variation in the normalized relative quantities (CV) were calculated. If a gene is stably expressed in a homogeneous sample, an M value < 0.5 and a CV < 25% should be seen.

#### 2.7.2. Assessment of Positive and Negative Controls

Several control samples were included in analyses for quality assurance. No template controls (NTCs) were used to detect the presence of contaminating DNA or the formation of primer dimers, determined by the difference in Cq value between the NTC and the investigated sample. The minus reverse transcription control (-RTC) was included to assure the absence of contaminating genomic DNA. An amplification signal in the -RTC was ignored when the difference in Cq value between the RTC and the sample with the highest Cq value was significantly larger. Biological controls against which up- or down-regulation was tested and where possible negative or positive controls were included.

#### 2.7.3. Deviating Sample Normalisation Factors

Normalization factor values for VRG should be similar for all samples when using approximately equal amounts of equal quality input material. The variability of normalization factors greater than 2- to 3-fold of the average is indicative of large differences in the starting material quantity or quality, or that a reference gene is not stably expressed or adequately measured.

### 2.8. Statistical Analysis

For statistical analysis, the Graphpad prism6 software package (GraphPad Software, Inc., La Jolla, CA, USA) was utilized. As there were multiple groups of differently treated samples, analysis of variance (ANOVA) was used to determine the differences across the groups. The statistical significance of the RT-qPCR results was assessed by the Repeated-Measure ANOVA with Tukey correction to evaluate the differences between means. This analysis was carried out on the Cq values after the calculation of the transcript copy number (fold change). The graphical representation of the RT-qPCR results represents the calculated transcript copy number, and the statistics shown in the graphs represent analysis based on the Cq values. The results were considered significant at *p* < 0.05.

## 3. Results

### 3.1. Cell Viability: Total Adenosine Triphosphate (ATP) and PrestoBlue Assays

Both CPZ and APAP did not compromise HepaRG cell viability at sub toxic concentration. Using total ATP and PrestoBlue assays, the effect of CPZ and APAP on HepaRG cells have already been published from our group [17,19].

### 3.2. RT-qPCR Efficiency

To compare the different RNA transcription levels, within and between experimental groups Cq (quantitation cycle) values of RT-qPCR reactions were compared directly. The Cq is defined as the number of cycles needed for the fluorescence signal to reach a specific threshold of detection and is inversely correlated with the amount of template nucleic acid present in the reaction [21]. Cq comparison relies on the RT-qPCR efficiency of primers for all RG and GOI (Gene of Interest) being comparable. This was calculated by the primer supplier (PrimerDesign) as between 90% and 100%, indicating that primers were suitable for the delta Cq method of data analysis.

### 3.3. RNA Transcription Levels of Putative Reference Genes in Various Experimental Settings

**Figure 1:**
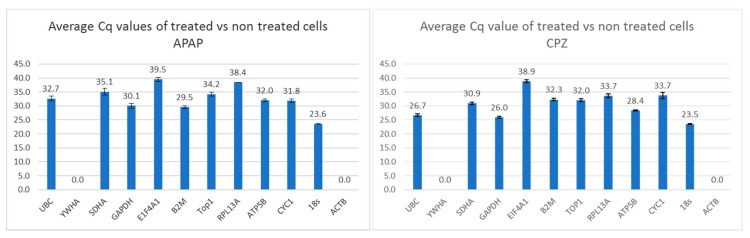
The RNA transcription of the tested reference genes in absolute Cq values over all investigated samples (cells stimulated with different doses of chlorpromazine (CPZ) or acetyl-para-aminophenol (APAP) and untreated cells). Values of Cq >40 are excluded.

Real-time RT-qPCR was used to measure the RNA transcription level of a panel of candidate housekeeping genes in HepaRG cells exposed to different concentrations of APAP and CPZ. To evaluate the stability of candidate reference genes, RNA transcription levels over all samples (untreated, drug-treated, and drug concentration) was measured (Figure 1). When genes were listed in order of expression in high (median Cq <30) or low (median Cq >30) expression groups, it was immediately apparent that for each drug tested the candidate genes in the high and low expression groups were different. Highly expressed genes in CPZ-treated samples included 18S, GAPDH, UBC, and ATP, whereas for APAP samples it included 18S and B2M. Genes with low expression following CPZ treatment included SDHA, B2M, TOP1, RPLI3A, CYC1 and EIF4A1, whereas following APAP treatment genes with low expression included UBC, SDHA, GAPDH, EIF4A1, TOP1, RPLI3A, ATP, and CYC1. Genes expressed at low levels or those not detectable in most samples (median Cq >40) were excluded from analyses by geNorm.

In general, we found that some genes were highly expressed, whereas others were only expressed at low levels or were not detectable in most samples. Following both CPZ and APAP treatment, the EIF4A1 gene showed the lowest RNA transcription level, whereas 18S was the most highly expressed gene (Figure 1). The YWHA and ACTB genes were not detectable in most samples (Figure 1).

The group of highly expressed genes (median Cq <30) in CPZ-treated samples included the following genes listed in the order of their RNA transcription levels: 18S, GAPDH, UBC, and ATP. The group of low expressed genes (median Cq >30) in CPZ-treated samples is as follows: SDHA, B2M, TOP1, RPLI3A, CYC1, EIF4A1. The group of highly expressed genes (median Cq <30) in APAP-treated samples included the following genes listed in the order of their RNA transcription levels: 18S and B2M. The group of low expressed genes (median Cq >30) in APAP-treated samples is as follows: GAPDH, CYC1, ATP5B, UBC, TOP1 SDHA, RPL13A and, EIF4A1. The genes expressed at low levels or those not detectable in most samples (median Cq >40) were excluded from analyses by GeNorm.

Based on using the same amount of cDNA in RT-qPCR reactions, the RNA transcription range was calculated as the difference between the lowest RNA transcription (high Cq value) and the highest RNA transcription (low Cq value) in all samples. The lowest RNA transcription range of an individual gene is a good indicator of constant RNA transcription over all samples.

For CPZ–treated cells, genes with a low range were Top1 (range = 2.0) then SDHA (range = 2.1), GAPDH (range = 2.3), UBC, and ATP (range = 2.4). For APAP treatment, the lowest RNA transcription range of an individual gene was for the ATP (range = 4.6) followed by CYC1 (range = 5.6).

In the CPZ group, the highest RNA transcription range of an individual gene, was for 18S (range = 10.7) and for APAP it was for UBC (range = 14.5). The transcription ranges for genes was completely different between the two drug treatments, as was the expression level.

### 3.4. Stability Analysis Per Experimental Condition

**Figure 2:**
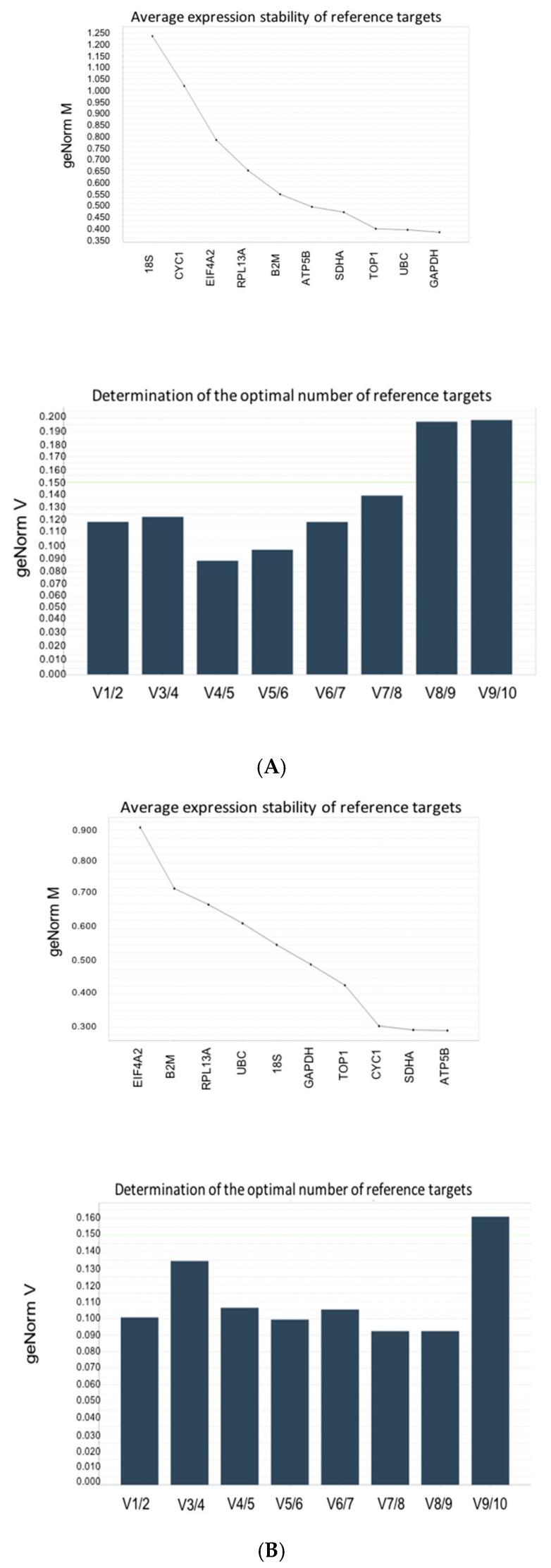

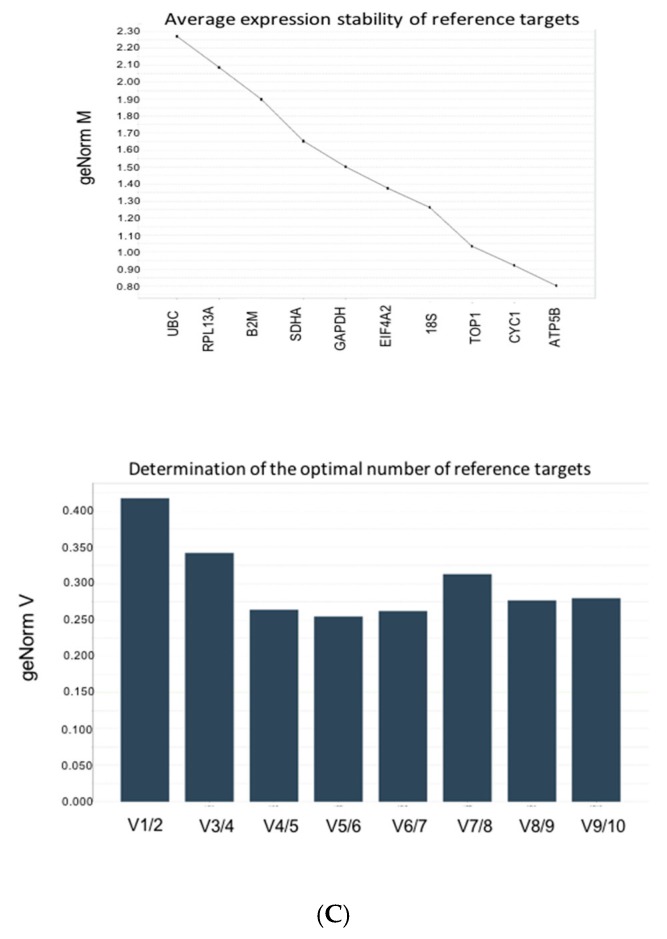
Optimal reference target selection. (**A**) M and V values for CPZ-treated and untreated cells. M and V values for CPZ-treated and untreated cells. The optimal number of reference targets in this experimental situation is 3 (geNorm V < 0.15 when comparing a normalization factor based on the 2 or 3 most stable targets). As such, the optimal normalization factor can be calculated as the geometric mean of reference targets GAPDH, UBC, and TOP1. (**B**) M and V values for APAP -treated and untreated cells. M and V values for APAP-treated and untreated cells. The optimal number of reference targets in this experimental setting is 3 (geNorm V < 0.15 when comparing a normalization factor based on the 2 or 3 most stable targets). As such, the optimal normalization factor can be calculated as the geometric mean of reference targets ATP5B, SDHA, and CYC1. (**C**) M and V values for combined CPZ- and APAP-treated and untreated cells. M and V values for combined CPZ- and APAP-treated and untreated cells. No optimal number of reference targets could be determined, as the variability between sequential normalization factors is relatively high (geNorm V > 0.15). Therefore, the optimal number of reference targets in this experimental setting is 5. As such, the optimal normalization factor can be calculated as the geometric mean of reference targets with the lowest M value (ATP5B, CYC1, TOP1, 18S, and EIF4A1).

geNorm analysis of all samples showed that, for different experimental conditions, a different set of reference genes was required. Applying the default criteria of the geNorm module in qbase+ 4 reference gene expression stability (M) and the number of reference genes (V) required for the normalisation of GOI data was determined for each drug by grouping samples according to experimental conditions. In each of these groups, the number of samples, which fulfilled the criteria for geNorm analysis, is given (Figure 2, Table 3).

The HepaRG cell line is a bipotent co-culture of hepatocytes and cholangiocyte-like cells, each of which may be differentially affected by changes during disease development or treatment with external compounds, which is why the identification of reference genes specific to experimental conditions is important for interpretation of data. When all data from both hepatotoxin treated groups at all concentrations and untreated controls were analysed as one set, none of the candidate genes reached an M-value below 0.5 and only two genes had an M-value below 1.0, indicating medium gene expression stability even in a homogeneous set of samples. This validates the argument that a single housekeeping gene should not be used to normalise data from different treatment groups.

No combination of genes from the panel reached a V-value below 0.15, and the optimal number of reference targets recommended by geNorm in this experimental situation is five. Although the reference genes are not optimal for normalisation as M > 1, using 3–5 genes with the lowest M value will result in a more accurate normalization as compared to using a single gene with the lowest M value.

Applying geNorm to data from the CPZ-treated cells identified five genes with an M-value below 0.5 and three genes with an M-value below 1.0, indicating good gene stability in a sample set treated with one compound (Figure 2A). The combination of three or more genes reached a V-value below 0.15. This represents the optimal number of reference targets to use for the normalisation of gene expression levels in samples treated with CPZ. Similarly, in APAP–treated HepaRG cells, a combination of three or more genes reached a V-value below 0.15. In this experimental setting, however, five of the reference genes reached an M-value below 0.5 and all other genes had an M-value below 1.0 (Figure 2B). As material was limited, a set of three appropriate genes were chosen to normalise data for each drug treatment. For CPZ: GAPDH, UBC and TOP1; for APAP: ATP5B, SDHA, and CYC1.

This requirement to use different gene sets for normalisation under the different experimental conditions may reflect the specific cellular targets of the pharmacological compounds causing changes in phenotypic and morphological profile.

When the experimental groups were combined (APA- and CPZ-treated and untreated cells), the stable expression of two genes (TOP1 and ATP5B) was seen. As the variability between sequential normalization factors was relatively high (geNorm V > 0.15), it would be advisable to use the five reference targets with the lowest M value for analysis across all experimental conditions. In this case, the geometric mean of ATP5B, CYC1, TOP1, 18S, and EIF4A1 expression should be optimal for normalisation across experimental settings (Figure 2C). This implies that if more than one pharmacological compound is tested simultaneously, a completely new geNorm analysis is required to select the optimal set of genes for data normalisation.

The expression of TOP1 and ATP5B was different between the two drug treatments. ATP5B was the most stable gene in samples treated with APAP (M < 0.3), whereas it was fifth in samples treated with CPZ (M < 0.5). Similarly, GAPDH expression was most stable in samples treated with CPZ (M = 0.375) but was fifth following treatment with APAP (M < 0.5). Although the three reference genes identified for normalisation were different for each experimental setting (APAP: SDHA, ATP5B, CYC1 and CPZ: GAPDH, UBC, TOP1) other genes with M < 0.5 could be included: TOP1 (in the CPZ panel) was the fourth most stable gene in APAP- (M = 0.425) treated cells and SDHA (in APAP panel) was the fourth most stable gene in CPZ-treated cells (M = 0.462).

### 3.5. Stability of RNA Transcription Following CPZ and APAP Stimulation (Data from CYP3A4 Analysis)

To investigate the stability of housekeeping gene transcription under experimental conditions, the Cq values of the geometric mean of geNorm-validated panels and classically used reference genes GAPDH and 18S were compared.

The average Cq value of validated reference genes for CPZ-treated cells (GAPDH, UBC, TOP1) was comparable at all concentrations tested. However, there were large variations in average Cq values at different CPZ concentrations for GAPDH and 18S genes, resulting in significant differences (Table 4). No significant differences were seen between the validated reference genes (VRG) (ATP5B, SDHA, and CYC1) for APAP-treated and untreated cells, and again there were large variations in the Cq of GAPDH and 18S. Applying geNorm to identify genes stably expressed across experimental conditions should improve the interpretation of RT-qPCR data.

### 3.6. Application of Identified Reference Genes

The normalisation of the expression of the genes of interest (CYP3A4 and HNF4α) was compared using classic reference genes (GAPDH and 18S) and using identified and validated appropriate reference gene sets. 

#### 3.6.1. Normalizing Data from CPZ-Treated HepaRG Cells

**Figure 3:**
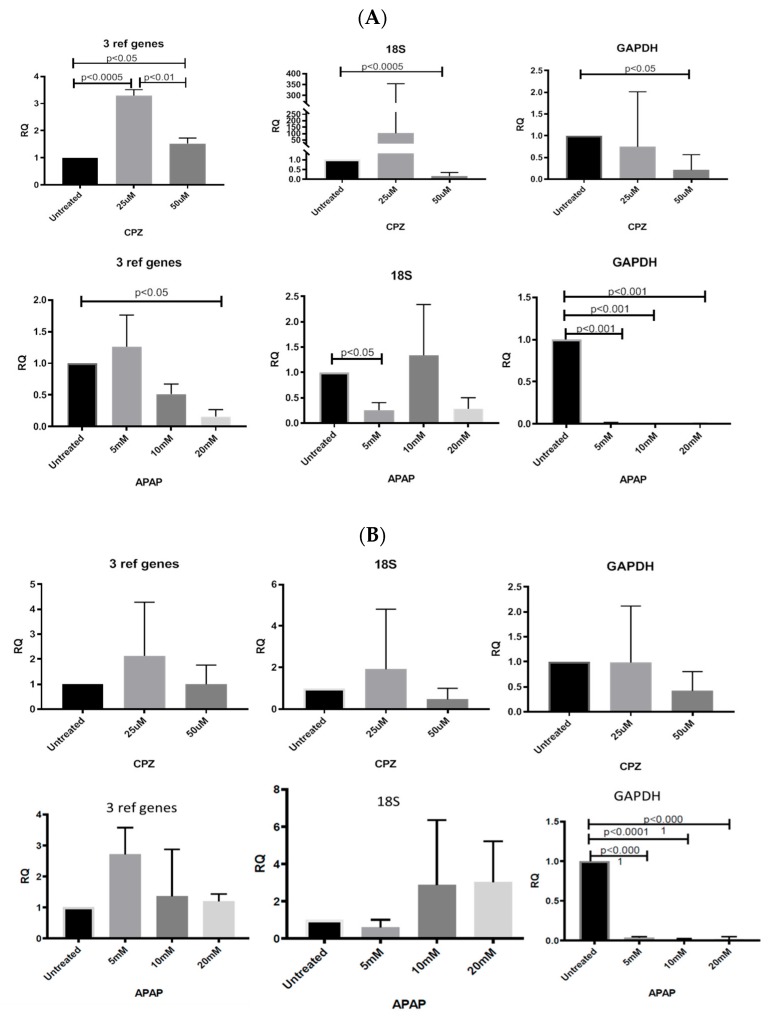
Fold change in the mRNA expression (±SD) of (**A**) CYP3A4 and (**B**) HNF4a in HepaRG cells stimulated with different doses of CPZ or APAP when mRNA expression was normalized using: the geometric mean of the validated reference genes (n = 4), 18S (n = 6), and GAPDH (n = 5). Fold change in mRNA expression is relative to HepaRG untreated cells. The application of single reference genes for data normalization show significant transcription discrepancies in comparison with data normalized with validated reference genes.

Data from CPZ-treated HepaRG cells with validated reference genes (GAPDH, UBC, TOP1) (Figure 3) showed expression of CYP3A4, one of the major metabolic enzymes within the liver, and was significantly upregulated by 3.5-fold at 25 μM CPZ, (*p* < 0.0005) and 1.5-fold at 50 μM CPZ, (*p* < 0.05) compared with untreated cells. This may be due to an attempt to detoxify the cellular environment. The lower expression of CYP3A4 expression at 50 μM CPZ (*p* < 0.01), compared with the 25 μM treatment, was possibly due to increased toxicity at the higher concentration. Using 18S alone for normalisation showed a ~100 fold up-regulation of CYP3A4 expression at 25 mM CPZ and downregulation at 50 mM compared to untreated cells. The M value of 18S (M = 1.137) was higher than for any other gene assessed in the panel for CPZ samples and associated instability of expression may account for the difference by comparison with the geNorm selected panel (Table 3). Normalisation to GAPDH showed no significant changes in CYP3A4 expression (Figure 3) with a downward trend in gene expression levels with increasing CPZ concentration. The expression of hepatocyte nuclear factor HNF4α was not significantly changed by CPZ treatment at either concentration as assessed by the geNorm panel and 18S or GAPDH. Changes in HNF4α, which directly regulates the CAR- and PXR-mediated expression of CYP3A4, showed some correlation with changes in CYP3A4 expression (Figure 3).

#### 3.6.2. Normalising Data for APAP-Treated HepaRG Cells

Data for APAP-treated HepaRG cells using geNorm-validated genes showed that CYP3A4 expression was significantly downregulated at 20 mM (*p* < 0.05) with a downward trend at 10 mM (Figure 3). Using 18S as a reference gene, 5 mM of APAP significantly attenuated CYP3A4 expression (*p* < 0.05) (Figure 3) but did not change significantly at higher APAP concentrations. Using GAPDH as a reference gene, CYP3A4 was significantly downregulated at all concentrations tested as compared to the untreated control. This was probably a result of increased GAPDH expression in treated cells as compared to the untreated controls. Normalising HNF4α expression to 18S showed non-significant upregulation at 10 and 20 mM (Figure 3). The large variations in average Cq values for single reference genes at different APAP concentrations resulted in significant discrepancies of the analysed data.

#### 3.6.3. Normalising Data for Low and Highly Abundant Liver Gene

RT-qPCR analysis was also applied to comparing the normalisation of the expression levels of low and highly abundant liver gene transcripts using VRG, GAPDH, or 18S. Albumin shows the highest RNA transcription level in liver20, and cyclin-dependent kinase inhibitor 2A (CDKN2A), an essential regulator of the cell cycle and cancer-related transcription factor, is not expressed in healthy hepatocytes.

**Figure 4:**
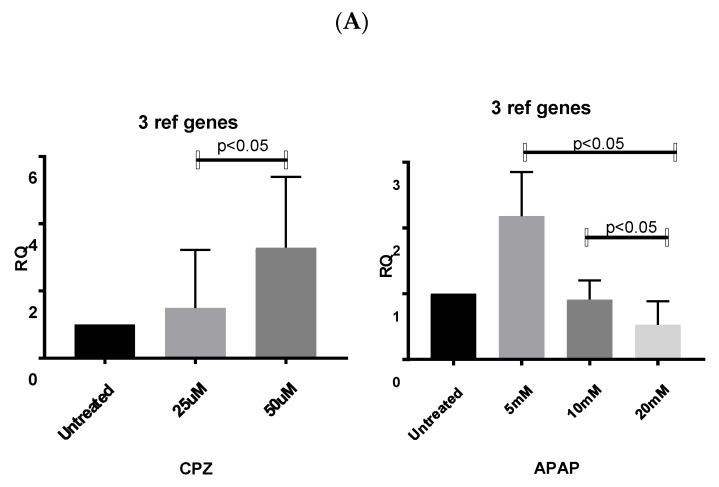

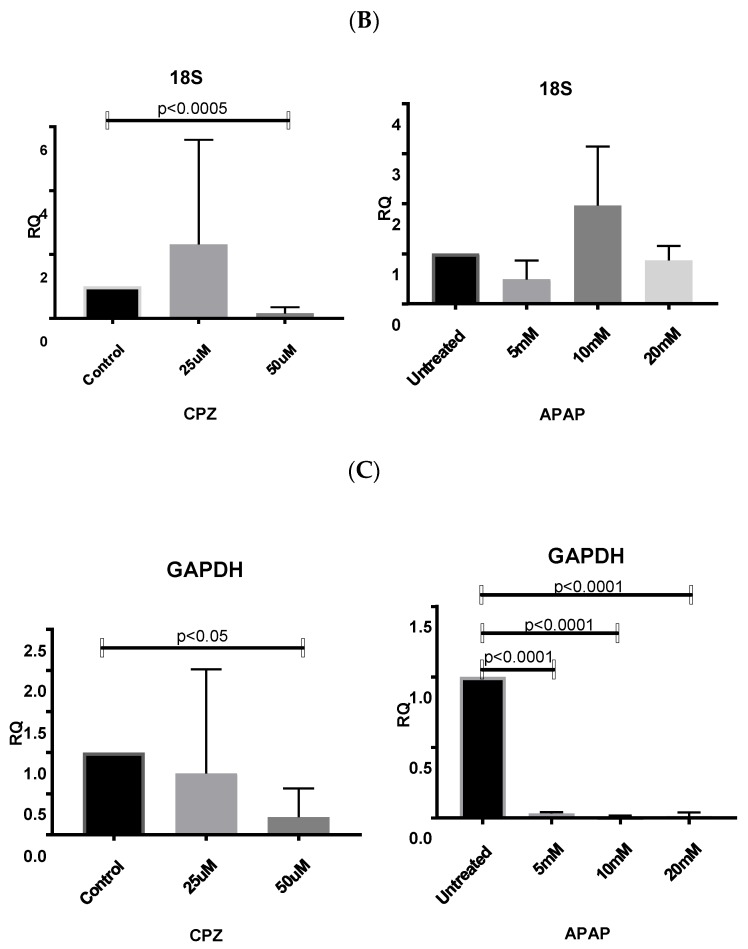
Fold change in mRNA expression (±SD) of liver abundantly expressed Albumin in HepaRG cells stimulated with different doses of CPZ or APAP when mRNA expression was normalized using: (**A**) the geometric mean of the validated reference genes (n = 5), (**B**) 18S (n = 6), and (**C**) GAPDH (n = 5). The fold change in mRNA expression is relative to HepaRG untreated cells.

Increasing CPZ concentration induced significant Albumin expression (*p* < 0.05) when results were normalized using VRG. Normalisation to 18S showed a trend in upregulation at 25 mM and downregulation at 50 mM (*p* < 0.0005). Using GAPDH showed the opposite result to that seen using VRG, with significant downregulation of albumin induced by 50 mM CPZ concentration (*p* < 0.05) (Figure 4).

APAP treatment showed significant (*p* < 0.05) albumin attenuation at 20 mM compared with 5 mM and 10 mM normalised using VRG. Using 18S showed no significant effect of APAP on albumin expression, while normalisation to GAPDH expression showed significant downregulation at all APAP doses when compared with untreated cells (5 mM *p* < 0.0001, 10 mM *p* < 0.0001, 20 mM *p* < 0.0001) (Figure 4).

**Figure 5:**
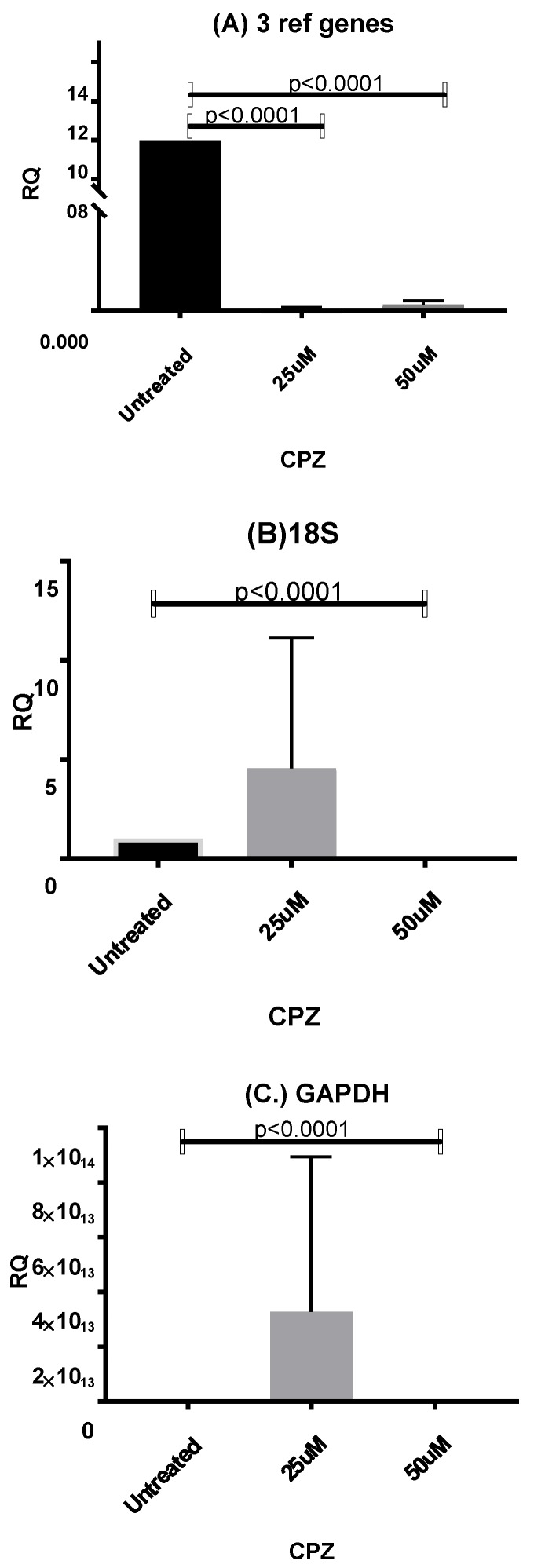
Fold change mRNA expression of senescence marker CDKN2A. The fold change in mRNA expression (±SD) of marginally expressed gene Cdkn in HepaRG cells stimulated with different doses of CPZ or APAP when mRNA expression was normalized using: (**A**) the geometric mean of the validated reference genes (n = 5), (**B**) 18S (n = 6), and (**C**) GAPDH (n = 5). The fold change of mRNA expression is relative to HepaRG untreated cells. CDKNA was not detectable in APAP-treated samples.

The expression of low abundance liver gene transcript CDKN2A, was significantly attenuated in all samples treated with CPZ when compared with the untreated control (25 μM *p* < 0.0001, 50 μM, *p* < 0.0001) when normalized using VRG, whereas analysis using single reference genes, GAPDH, and 18S only showed significant downregulation of CDKN2A in cells treated with (50 mM, *p* < 0.0001) (Figure 5). The insignificant upregulation of CDKN2A in cells treated with (25 mM) can be explained by high GAPDH and 18S RNA transcription regulation, depicted by the large variations of single reference gene Cq values responsible for high standard deviations within investigated groups. CDKN2A was not detected in samples treated with APAP.

These analyses showed that the application of single reference genes, e.g., GAPDH or 18S, results in significant GOI RNA transcription differences that could even vary 100-fold, especially for low abundance gene transcripts as compared to using the geNorm-validated panel.

## 4. Discussion

In molecular biology, RT-qPCR is used as a “gold standard” for gene expression studies. However, there is controversy regarding the reproducibility and accuracy of results generated using this technique [1]. Changes in the expression of a gene of interest are calculated by comparison with a gene whose expression is not changed by the experimental conditions, i.e., a housekeeping or RG. Common limitations of RT-qPCR experiments are the RG selected and the number of RGs that are used to normalize RT-qPCR data. It has become well-established that the use of more than one RG improves accuracy compared to the use of a single RG, especially when the aim is to show relatively low fold changes in RNA levels [5,22,23]. For large-scale studies, especially relevant in predictive toxicological research, using mean expression values of stable genes identified using the geNorm tool is recommended as the best normalization strategy [24].

Application of the highly functional and differentiated cell line HepaRG to hepatology research is gaining global importance as a human-relevant source of hepatic cells [12]. The human bipotential progenitor HepaRG cell line has a long life span in vitro and gives phenotypically and functionally consistent data. These are advantages over PHHs, which have a short in vitro life span and donor variability. HepaRG cells are a highly differentiated hepatocyte:cholangiocyte, co-culture model, which maintain liver-specific functions at levels comparable with PHHs. We have previously shown that hepatic cell lines can be successfully used for liver disease modelling [25,26]. The increasing use of in vitro cellular models for early phase drug tests and disease modelling requires a comprehensive analysis of normalization methods for the evaluation of RT-qPCR experiments to ensure consistent interpretation of results.

Most pharmacological compounds modulate key cellular processes, which may alter the expression of currently used RT-qPCR reference genes, as hepatotoxins affect different cellular transcription pathways in different ways. Thus, it is not possible to identify a single housekeeping gene for use in the RT-qPCR studies of all compounds under test. To date, the variability of housekeeping gene expression has been studied in HepaRG progenitor cells undergoing differentiation [12]. However, to our knowledge, such a study has not been carried out on fully differentiated HepaRG cells used as a model for hepatotoxin testing. We have extended the published investigations by applying the geNorm tool to determine the suitability of 12 RGs for the analysis of gene expression by a differentiated HepaRG human hepatic cell line treated with a range of concentrations of APAP or CPZ, two hepatotoxins with different mechanisms of action.

The geNorm analysis of all samples, APAP- and CPZ-treated, at all concentrations and untreated cells showed that the commonly used house-keeping RG genes, 18S and GAPDH, were not stably expressed across all treatments and therefore were ranked by the geNorm software as unsuitable as candidates for HepaRG transcriptional analysis unless used in a set of RGs (Figure 2). Both 18S and GAPDH have been commonly used as single internal transcriptional controls: in the vast majority of studies using HepaRG cells, normalisation to 18S expression has been used without the verification of expression stability under different conditions [27,28].

Our results show that stable RNA transcription for CPZ or APAP treatment is only met by three selected RGs: (CPZ: GAPDH, UBC, and TOP1) or (APAP: ATP5B, SDHA, and CYC1). Our results also show that the expression of GAPDH and 18S is not consistent between experimental settings (Table 4. geNorm analysis additionally showed that 18S was the least stably expressed reference gene in HepaRG cells treated with CPZ and the sixth stably expressed reference gene in APAP-treated samples. In addition, it is of note that although GAPDH was one of the most reliable reference genes identified in the CPZ samples, it was the most 5th suitable candidate in the APAP samples. These findings highlight the importance of evaluating the choice of housekeeping genes and emphasize the importance of selecting of a set of RGs for normalisation to address variability induced by different experimental treatments within a single cell type.

The identification of stable RGs for use in the RT-qPCR analysis of CPZ and APAP treatments allowed us to normalize and compare the expression level of selected liver target genes Cyp3A4 and HNF4α using the geometric mean of validated reference targets as well as the classically used RGs GAPDH and 18S. The expression of CYP3a4 and HNF4α differed between drug treatments after normalizing with the geometric mean of specific VRG (Figure 3). The analysis of expression based on normalizing the expression of GOI using only GAPDH or 18S gave results different from those generated by normalizing using the geometric mean of validated reference targets. Such results are in accordance with those reported previously [29].

In this study, in a group of CPZ–treated samples, our results clearly demonstrated no significant difference between the low and high dose of CPZ as well as between 25 μM CPZ–treated cells and untreated cells when GAPDH alone was used for normalisation (Figure 3). Although GAPDH was one of the most reliable RGs identified in this group, variation in Cq between groups was noted (Table 4). Similarly, using 18S as RG, there was no significant difference between 25 μM CPZ–treated cells and untreated cells (Figure 3) and the fold change in the group of cells treated with 25 μM CPZ exceeded 100 when compared with the untreated cells. These examples clearly indicate that these genes were not stably expressed under drug stimulation and therefore a single reference should not be used to normalise the transcription level of GOI within a cell line under drug stimulation.

Applying GAPDH ranked as the 5th suitable candidate for the data normalisation of APAP-treated cells, CYP3A4 and HNF4α mRNA expression were significantly attenuated for all treatments. Using 18S, the sixth stably expressed reference gene in APAP samples only showed the significant downregulation of CYP3A4 at the lowest dose. HNF4α mRNA expression was not significantly changed and there was no correlation between CYP3A4 and HNF4α mRNA expression. However, when mRNA expression was normalized using the VRG, both genes showed a similar response to the drug treatment and 20 mM APAP significantly attenuated CYP3A4 expression when compared with untreated cells.

CYP3A4 subfamily enzymes play a major role in the metabolism of ~30% of clinically used drugs from almost all therapeutic categories [30,31,32]. As previously reported [33], this major P450 enzyme is involved in CPZ oxidative metabolism, which is active in CPZ-treated in HepaRG cells. This result, together with reports of CPZ-inducing CYP3A4 activity in vivo, agrees with the results from our study when gene expression was normalised using geNorm-validated reference genes. Using the geNorm-validated panel of reference genes, we found no upregulation of CYP3A4 following APAP treatment samples, also reported following a single APAP treatment in vivo [34].

Comparing the expression level of low and high abundance liver gene transcripts CDKN2A and albumin respectively, normalisation to single reference genes, GAPDH, or 18S showed variation in transcription. In the case of low abundance gene transcripts CDKN2A, this reached up to 100-fold. This emphasizes the need to identify candidate RGs in HepaRG cells specific to experimental conditions as reference genes are differently regulated under different conditions.

Collectively, these data showed that using GAPDH or 18S as a single RG for normalisation gene expression in HepaRG cells may result in misinterpretation of RT-qPCR data.

Several studies have reported that expression levels of GAPDH are highly variable and dependent on experimental conditions. Therefore, its use as a reference would not be optimal for use when comparing changes in gene expression associated with induction of differentiation over time [35]. This finding has been reported from studies of differentiation of human adipose-derived mesenchymal stem cells [36], from human liver samples [37], in non-alcoholic fatty liver disease (NAFLD) animal models [38], different pig tissues [39], and plant samples [40]. Those reports show that when GAPDH was used as an internal control, the investigated GOI had no significant change or had fluctuating patterns of expression, indicating that GAPDH is unreliable for RT-qPCR analysis. Earlier published data also demonstrated that GAPDH and 18S displayed variable expression levels in models of hepatitis C virus infections [41] and in patients with an alcohol-induced liver injury [42]. In contrast, another study showed that GAPDH, eEF-1, and UBC were suitable reference genes for porcine dorsal root ganglia samples, whereas ACTB, SDHA, and UBC were more appropriate for spinal cord samples [43].

In conclusion, we assessed the expression stability of 12 commonly used RGs. Future studies may consider different gene combinations, including the TPB gene or other well characterised genes for metabolic function. We have demonstrated that application of 18S or GAPDH commonly used as RGs for normalisation of RT-qPCR data were consistently unreliable and should be used with caution as individual genes in studies involving HepaRG cells. Testing a panel of 12 RGs by using the geNorm tool to validate expression stability can identify as few as three RGs to serve as a good standard for normalizing RT-qPCR data from differentiated HepaRG cells exposed to hepatotoxins, such as CPZ and APAP. Each hepatotoxin required a different set of RGs for normalisation. These selected RGs showed high stability in untreated and drug-treated HepaRG cells. To avoid misinterpretation of RT-qPCR data in hepatotoxicity testing, using HepaRG or other cell lines, specific reference genes should be identified to avoid misinterpretation of data. This is also an important consideration in the field of clinical cell therapy using differentiated stem cells, where accurate monitoring of dynamic sequential changes in gene expression are vital to assuring quality control and safety.

## Figures and Tables

**Table 1:** Primer sequences for the custom real-time PCR (Primerdesign Ltd., Southampton, UK).

Gene Symbol	Gene Name	Forward Primer	Reverse Primer	Amplicon
**ALB**	Albumin	TGACAAATCACTTCATACCCTTTTT	GCATTCATTTCTCTCAGGTTCTTG	118
**CYP3*A*4**	Cytochrome P450 family 3 subfamily A member 4	ACCGTAAGTGGAGCCTGAAT	AAGTAATTTGAGGTCTCTGGTGTT	90
**HNF4α**	Hepatocyte nuclear factor 4α	GACCTCTACTGCCTTGGACAA	GATGAAGTCGGGGGTTGGA	87
**CDKNA**	Cycli-dependent kinase inhibitor 2A	GGAAGGTCCCTCAGACATCC	CTTCGGTGACTGATGATCTAAGTT	84

**Table 2:** The 12 housekeeping gene candidates and function. The genes function description is provided based on the information from the human genome database (GDB, http://www.gdb.org).

Gene Name	Gene Function
**GAPDH (glyceralde- hyde-3-phosphate dehydrogenase)**	Product catalyses a step during carbohydrate metabolism, also has uracil DNA glycosylase activity in the nucleus, and contains peptide involved in antimicrobial activity.
**RPL13a (ribosomal protein L13a)**	Eukaryotic cytoplasmic ribosomal subunit.
**18S (18S ribosomal RNA subunit)**	Eukaryotic cytoplasmic ribosomal subunit.
**TOP1 (topoisomerase 1)**	Encodes a DNA topoisomerase, an enzyme that controls and alters the orientation of DNA during transcription.
**ATP5b (ATP synthase subunit 5b)**	Encodes a subunit of mitochondrial ATP synthase.
**UBC (ubiquitin C)**	Encodes a polyubiquitin precursor.
**SDHA (succinate dehydrogenase complex, subunit A)**	Encodes major catalytic subunit of the mitochondrial respiratory chain.
**B2M (beta-2 microglobulin)**	Encodes a serum protein found on the surface of most nucleated cells.
**β-Actin (beta-actin)**	Encodes one of six different actin proteins, involved in cell motility, integrity, structure, and intercellular signalling.
**CYC1 (cyclin D1)**	Role in cell proliferation.
**EiIF4a2 (eukaryotic initiation factor 4a2)**	Regulates lipid metabolism, and translation factors.
**YWHAZ (phospholipase A2)**	Encodes mediator of signal transduction.

**Table 3:** Stability values per gene in various experimental settings.

Experiment	All Conditions	APAP	CPZ
Samples Types	Number of samples		
Treated	5	6	6
Untreated	5	6	6
Reference Genes			
Gene	M-value		
UBC	2.262	0.6125	0.387
YWHA			
SDHA	1.9	0.287	0.462
GAPDH	1.50	0.487	0.375
EIF4A1	1.362	0.912	0.775
B2M	1.65	0.725	0.567
TOP1	1.012	0.425	0.387
RPL13A	2.062	0.675	0.65
ATP5B	0.8	0.287	0.487
CYC1	0.912	0.3	1.012
18S	1.25	0.55	1.137
ACTB			
Average M	1.0672	0.291333	0.383
CV	0.19604	0.021035	0.01477
Required number of genes	5	3	3

Notes: A. Grouping of categories of samples (rows) into experimental settings (columns). Per category, the number of included samples (N) are given. B. The reference genes required for normalization per experimental condition are indicated in gray; the M-values indicate the stability of the individual candidate reference genes in the experimental conditions. The average M-value and coefficient of variation (CV) are given for the required reference genes per experiment.

**Table 4:** RNA transcription level under CPZ or APAP stimulation for selected reference genes: (CPZ: GAPDH, UBC, and TOP1) or (APAP: ATP5B, SDHA, and CYC1) and single GAPDH or 18S genes.

	**Average Cq Values**		
**Untreated**	**GAPDH,UBC,TOP1**	**GAPDH**	**18S**
	32.4	31.2	24.4
	31.2	31.5	22.7
	31.1	33.7	25.8
average	**31.6**	**32.1**	**24.4**
st.dev	**0.6**	**1.1**	**1.1**
**25 μM CPZ**			
	33.5	31.10	22.9
	35.3	nd	24.1
	34.0	33.45	27.0
	35.3	22.63	10.4
	nd	22.29	11.5
	30.2	22.19	36.8
average	**33.6**	**26.3**	**22.1**
st.dev	**1.9**	**4.9**	**9.1**
**50 μM CPZ**			
	34.0	33.1	24.4
	34.5	30.8	24.2
	34.9		22.2
	34.9	26.1	10.9
	28.5	28.3	10.8
	30.2	29.0	14.7
average	**32.8**	**29.4**	**17.9**
st.dev	**0.4**	**2.3**	**5.9**
	**Average Cq Values**		
**Untreated**	**ATP,SDHA,CYC1**	**GAPDH**	**18S**
	26.7	31.2	22.7
	26.4	31.5	25.8
	27.3	33.7	24.8
average	**26.8**	**32.1**	**24.4**
st.dev	**0.4**	**1.1**	**1.3**
**5 mM APAP**			
	31.6	26.5	21.5
	32.2	27.6	23.9
	32.4	27.9	24.6
average	**32.1**	**27.3**	**23.3**
st.dev	**0.4**	**0.6**	**1.4**
**10 mM APAP**			
	31.6	27.1	24.9
	32.2	27.1	27.4
	31.4	26.7	27.8
average	**31.7**	**27.0**	**26.7**
st.dev	**0.3**	**0.2**	**1.3**
**20 mM APAP**	31.6	28.9	24.9
	32.2	24.0	27.4
	31.4	23.7	27.8
average	**31.7**	**25.6**	**26.7**
st.dev	**0.3**	**2.4**	**1.3**

Note: Variation of Cq values within investigated groups is depicted by standard deviations.
